# Ultrasound-guided high-intensity focused ultrasound treatment of breast fibroadenoma—a multicenter experience

**DOI:** 10.1186/s40349-014-0022-3

**Published:** 2015-01-22

**Authors:** Roussanka Kovatcheva, Jean-Noël Guglielmina, Marc Abehsera, Loïc Boulanger, Nicolas Laurent, Edouard Poncelet

**Affiliations:** Department of Thyroid and Metabolic Bone Disorders, University Hospital of Endocrinology, Medical University of Sofia, 2 Zdrave Street, 1431 Sofia, Bulgaria; Department of Gynecological Surgery, American Hospital of Paris, 63 Bld Victor Hugo, 92200 Neuilly-sur-Seine, France; Department of Medical Imaging, American Hospital of Paris, 63 Bld Victor Hugo, 92200 Neuilly-sur-Seine, France; Department of Gynecological and Breast Surgery, University Hospital of Lille, Ave Eugène Avinée, 59037 Lille, France; Department of Imaging, Regional Hospital of Valenciennes, Ave Désandrouin - BP 479, 59322 Valenciennes, France

**Keywords:** Breast fibroadenoma, Ultrasound (US)-guided high-intensity focused ultrasound (HIFU), Minimally invasive treatment

## Abstract

**Background:**

The aim of our multicenter study was to assess the clinical outcome and safety of ultrasound (US)-guided high-intensity focused ultrasound (HIFU) in patients with breast fibroadenoma (FA).

**Methods:**

From May 2011 to February 2013, 42 women with 51 FA in one or both breasts were selected for treatment with US-guided HIFU. Eight of 51 FA were treated twice. Patients’ age ranged from 16 to 52 years (mean 32 years). All patients with FA underwent core needle biopsy with histological confirmation. HIFU treatment was performed as an outpatient procedure under conscious sedation. Exclusion criteria were pregnant or lactating women, microcalcifications within the lesion at mammogram, history of breast cancer, previous laser or radiation therapy, and breast implant in the same breast. All patients signed written informed consent. After the treatment, follow-up US with volume evaluation was performed at 2, 6, and 12 months.

**Results:**

The FA mean baseline volume was 3.89 ml (0.34–19.66 ml). At 2-month follow-up, the mean volume reduction was 33.2% ± 19.1% and achieved significance at 6-month (59.2% ± 18.2%, *p* < 0.001) and 12-month (72.5% ± 16.7%, *p* < 0.001) follow-up. Related side effects as superficial skin burn with blister-like aspect in three patients and hyperpigmentation over the treated area in one patient were transient and resolved spontaneously. In one patient, asymptomatic subcutaneous induration persisted at the end of the study.

**Conclusions:**

US-guided HIFU treatment is an effective noninvasive method for the treatment of breast FA and well tolerated by the patients. Preliminary results are encouraging and show that HIFU could be an alternative to surgery for breast FA.

## Background

Breast fibroadenoma (FA) is a benign tumor, most often detected during self-examination or clinical breast examination. Usually occurring in women under the age of 30, they are seen in approximately 10% of all women during their lifetime [1,2]. FA account for between 30% and 75% of all breast biopsies, depending on the age of the population being sampled [3]. The widespread implementation of routine screening mammography has resulted in the fact that benign breast diseases represent a growing percentage of pathological findings [4].

The most accurate approach to the nonsurgical evaluation of clinically benign dominant breast masses in young women is the combination of clinical breast examination, breast imaging studies, and needle biopsy. Used together, these three modalities are commonly referred to as the triple test [5]. Ultrasound (US) is the method of choice in young women, whereas in women over 35, it has to be combined with mammography. Percutaneous core needle biopsy (CNB) is the most accurate tool for establishing the diagnosis [6]. The American Society of Breast Surgeons guideline indicates that US-guided percutaneous biopsy is the diagnostic procedure of choice for US-visible lesions.

Because of its superficial location, breast FA is especially suitable for minimally invasive ablation techniques. These techniques can be divided into heat-based modalities, which include high-intensity focused ultrasound (HIFU), radiofrequency ablation, laser ablation, and tissue-freezing technique referred to as cryoablation [7-12]. There are nonthermal ablative techniques as well, like vacuum-assisted biopsy [13]. US-guided HIFU is a noninvasive treatment method, without needle or probe insertion into the target, compared to the most promising image-guided minimally invasive procedures as vacuum-assisted biopsy and cryoablation [14,15]. In the last 10 years, the feasibility and the safety of HIFU have been tested in a number of clinical studies on benign and malignant tumors of the prostate, uterus, thyroid gland, parathyroid, liver, kidney, pancreas, bone, and brain [16-19].

The aim of our multicenter study was to assess the clinical outcome and safety of US-guided HIFU in patients with breast FA.

## Methods

### Study design

The study was performed in four centers—three in France (American Hospital of Paris, University Hospital of Lille, and Local Regional Hospital of Valenciennes) and one in Bulgaria (Sofia University Hospital of Endocrinology)—as uncontrolled, open label, and prospective. Patients over 18 years of age with at least one symptomatic breast FA, who met the protocol’s eligibility criteria, were recruited. The treatment was performed as an outpatient procedure, and follow-up visits were scheduled at 1 week, 2 months, 6 months, and 12 months after the last HIFU treatment. Adverse events during and after the procedure were evaluated.

### Patients

From May 2011 to February 2013, 42 women with 51 FA in one or both breasts were approved for treatment with US-guided HIFU. In five patients, more than one FA was selected: two patients with one FA in each breast, two patients with three FA (two in one breast and one in the other), and one patient with four FA (three in one breast and one in the other). Eleven subjects had a personal history of breast surgery for FA. The patients’ age ranged from 16 to 52 years (mean 32 years). A formal derogation was obtained in order to treat one 16-year-old patient.

Selection criteria included female patients ≥18 years with a palpable and US-visible breast FA, a negative mammogram for microcalcifications in patients over 35 years of age (Breast Imaging Reporting and Database System (Bi-RADS) score ≤2), and CNB with histological confirmation for FA by two independent readers. The technical criteria included the following: 1) FA size and distance behind the focal point ≥10 mm, to eliminate exteriorization of the focal energy and damage of vulnerable structures behind it; 2) distance between the skin and the FA anterior border ≥5 mm, to prevent skin burn; and 3) distance between the skin and the FA posterior border ≤23 mm, as a limit of accessibility for treatment with HIFU. These criteria had to be reached under treatment conditions, with an immobilized and potentially compressed breast. Patients with microcalcifications at mammogram and Bi-RADS score >2, history of breast cancer, previous laser or radiation therapy to the same breast, breast implants and pregnant or lactating women were excluded. FA located behind the nipple were also excluded if there was no technical possibility to avoid the nipple. The study was approved by the local ethics committees, and written informed consent was obtained from all patients.

### Imaging evaluation

FA visualization was performed with high-resolution real-time ultrasonography and color Doppler, using a 7.5- to 10-MHz linear transducer. The FA size was measured in two orthogonal planes—radial to the nipple and antiradial—and the volume was calculated according to the following formula: length × width × depth × *π*/6. Assessment of US characteristics like echogenicity (defined as anechoic, hypoechoic, isoechoic, and hyperechoic, related to the normal breast parenchyma), presence of macrocalcifications, and type of vascularization (peripheral and central, qualified as present or absent) was performed. FA localization was documented clock face.

After the HIFU treatment, physical examination was performed at 1 week and US follow-up at 2, 6, and 12 months to evaluate the changes of volume, echogenicity, and vascularization. In cases of volume reduction ≤50% and/or persisting symptoms after the 3-month visit, a second HIFU treatment was performed with the same follow-up.

### Equipment

**Figure 1:**
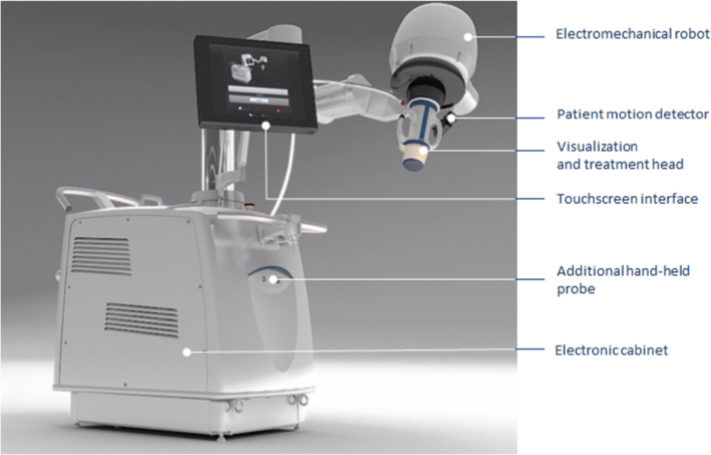
**External view of the US-guided HIFU equipment.**

**Figure 2:**
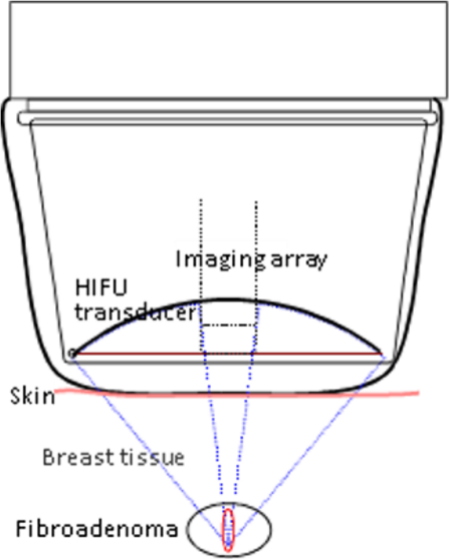
**Visualization treatment unit (VTU) components and principles of function.**

**Figure 3:**
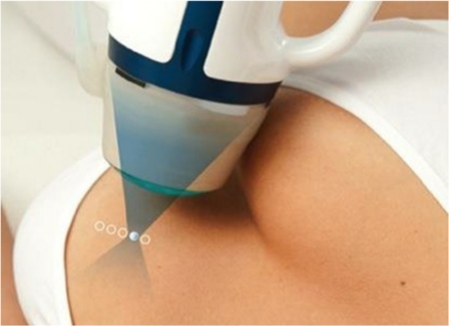
**Positioning of the treatment device (VTU) over the targeted fibroadenoma.**

The treatment was performed with an US-guided HIFU system, CE marked for this indication (EchoPulse, Theraclion, Paris, France) (Figure 1). The energy was delivered via an extra-corporal treatment probe which includes an imaging system (visualization treatment unit, VTU) (Figure 2). The high-intensity US waves propagated through the skin and were focused on a portion of the target tissue, generating rise of heat to therapeutic levels and causing thermal tissue ablation within the focused area (Figure 3). The HIFU-induced tissue changes were visible as hyperechoic marks. HIFU energy was delivered by the VTU device, which moved automatically over the defined treatment volume. As a result of the thermal damage, subsequent fibrosis and volume shrinkage developed, without effect on the surrounding parenchyma.

### Treatment procedure

The treatment session consisted of five steps: pretreatment ultrasonography, positioning, planning, energy setting, and treatment with HIFU pulses in the defined volume.

The patient was placed in lateral decubitus position, depending on the site of the targeted FA, with the arm positioned over the patient’s head. The breast was then immobilized with a specially designed mechanical device (Theraclion, Paris, France), and the patient was administered intravenously fentanyl or midazolam in combination with propofol to achieve conscious sedation. If the patient still had pain, a ketoprofen infusion was additionally applied.

**Figure 4:**
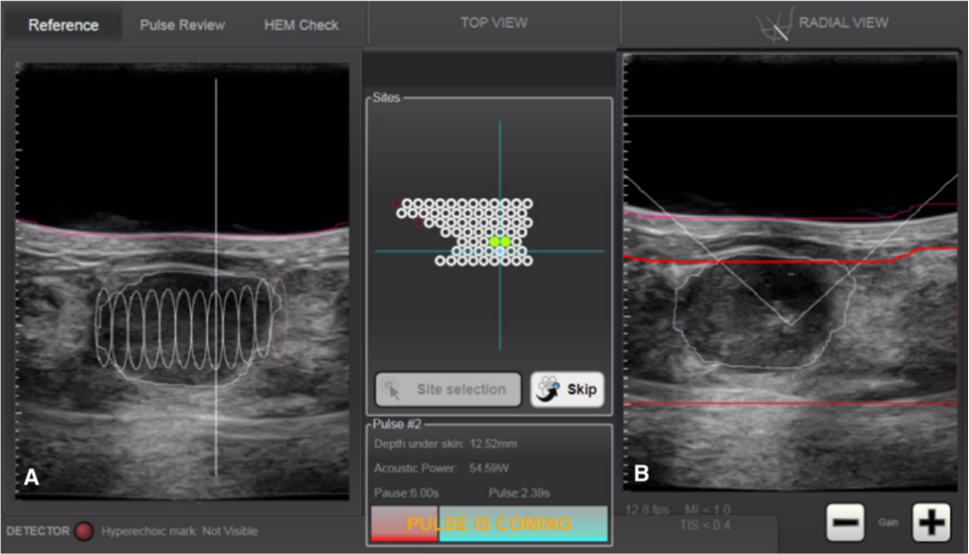
**Touch screen interface of US-guided HIFU system. (A)** The FA is outlined in radial US scan, and the planned treatment units are displayed on the screen. **(B)** Hyperechoic mark is visible at the point of the treatment cone after the sonication.

The physician outlined the FA on consecutive sections in radial and antiradial US scans and defined the position of the focal zone relative to the FA center (Figure 4A).Thereafter, the number of the treatment units and the time for treatment were automatically calculated, and the procedure started with a preselected energy level. During the treatment, the energy was individually adjusted in order to obtain hyperechoic marks on the US image as an indirect sign of thermal tissue damage (Figure 4B). Then, the procedure continued with repeated HIFU pulses to cover the whole treatment volume, under the physician’s control. The duration of the HIFU session depended on the FA size. At the end of the treatment, US examination with a color Doppler was performed.

After the procedure, all patients remained for at least 2 h in a recovery room to be monitored for posttreatment pain or side effects. An iced pad was placed over the treated FA; ibuprofen one or two tablets per day was recommended in case of pain in the next 24–48 h.

The patients were evaluated for side effects occurring during and after the treatment. Pain was assessed at the end of the procedure, using a 0- to 100-mm visual analog scale (VAS). A questionnaire about discomfort and pain related to the FA before and after the treatment was completed by all patients.

The effect of HIFU treatment was evaluated according to the changes in FA volume and improvement in clinical symptoms, including size by palpation and cosmetic concerns compared to baseline.

### Statistical analysis

All statistical analyses were carried out using SAS® version 9.2 under the Windows® 2008 terminal. A mixed model (MIXED procedure, SAS®) was used to estimate breast FA volume reduction at 6 and 12 months, and a logistic model (GLIMMIX procedure, SAS®) was used to assess the rate of successful volume reduction from baseline to 6 and 12 months. The relation between energy parameters and volume reduction was examined using Pearson correlations and mixed models. For subjects who underwent a second HIFU treatment, a generalized linear model (GLM procedure, SAS®) was used to estimate the rate of volume reduction from baseline obtained before and after the second HIFU treatment.

All models take into account possible multiple breast FA within subjects. All statistical tests were two sided at an alpha level of 0.05.

## Results

Forty-three FA (84.3%) were treated with a single HIFU procedure. The patient with four FA and one of the patients with three FA were treated in two sessions: one session for the lumps in the same breast, which were neighboring and up to 20 mm in size, and a second session for the lump in the other breast. All the other multiple lumps, found in one patient, were treated in separate sessions.

Eight FA (15.7%) underwent a second HIFU treatment between the 3-month and 9-month follow-up. The average treatment duration was 118 min, ranging from 60 to 255 min.

**Figure 5:**
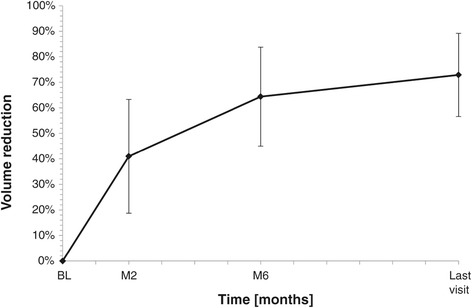
**Percentage of fibroadenoma volume reduction during the follow-up after US-guided HIFU treatment.**

**Figure 6:**
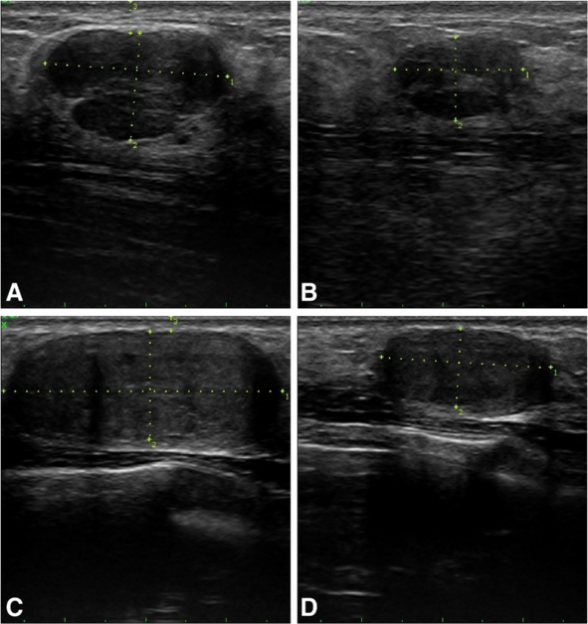
**Grey-scale US images of two breast fibroadenomas in antiradial scan, before and 12 months after US-guided HIFU treatment. (A)** A 24-year-old female patient with right breast FA at 2 o’clock and with 2.96 ml of initial volume. **(B)** Twelve months after a single HIFU treatment; 62.3% of volume reduction was obtained. **(C)** Right breast FA at 7 o’clock, with 8.14 ml of initial volume in the same patient. **(D)** Twelve months after two HIFU treatments; the FA volume reduced by 73.5%.

The mean FA volume at baseline was 3.89 ml (0.34–19.66 ml). At 2-month follow-up, the mean volume reduction was 33.2% ± 19.1% and achieved significance at 6-month (59.2% ± 18.2%, *p* < 0.001) and 12-month (72.5% ± 16.7%, *p* < 0.001) follow-up (Figure 5). A volume decrease of 30% at 2-month, 50% at 6-month, and 60% at 12-month follow-up was present in 63% (29/46), 67% (32/48), and 87% (40/46) of cases, respectively (Figure 6).

At baseline US, the echogenicity was hypoechoic in 90%, anechoic in 4%, hyperechoic in 2%, and complex in 4% of FA. No significant change in the echogenicity was observed during the follow-up. Color Doppler signals were absent in 39% of FA at baseline. Two months after HIFU treatment, vascularization was absent in 54% of FA and this percentage increased up to 64% and 67% at 6-month and 12-month follow-up, respectively.

**Figure 7:**
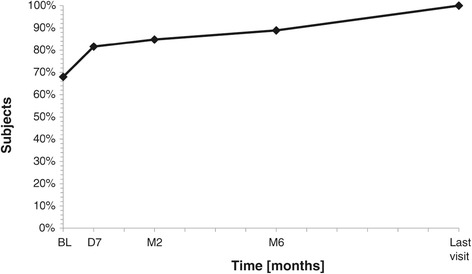
**Percentage of subjects without discomfort in daily activities before and after US-guided HIFU treatment.** *BL* baseline, *D7* 7 days, *M2* 2 months, *M6* 6 months.

**Figure 8:**
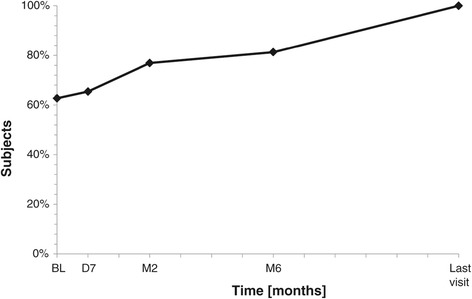
**Percentage of subjects without pain related to the fibroadenoma before and after US-guided HIFU treatment.** *BL* baseline, *D7* 7 days, *M2* 2 months, *M6* 6 months.

All but one patient (98%) were presenting a palpable breast mass. Discomfort due to the FA was mentioned in 31 cases (60.8%), which include 15 cases (29.4%) with discomfort during daily activities, ten cases (19.6%) during sports activities, and six cases (11.8%) during professional activities. Pain related to FA was documented in 18 cases (35.3%). The cosmetic appearance was modified in ten cases (19.6%), and anxiety due to the presence of FA was reported in 29 cases (56.9%). A continuous reduction in the discomfort in daily activities, perceived by the patients, was observed during the follow-up period and completely disappeared in all cases after 12 months (Figure 7). Pain related to the FA at baseline progressively decreased through the follow-up and totally disappeared (Figure 8).

### Safety evaluation

All the patients tolerated well the HIFU treatment, and no serious adverse event was reported.

The mean VAS score was 29.7 ± 27.5 mm (range 0–80 mm), and no one needed analgesic drugs immediately after the treatment. Superficial skin burn with blister-like aspect in three cases (5.9%) and hyperpigmentation over the treated skin area in one case (2%) were described. In one case, a palpable subcutaneous induration between the FA and the skin was found. The changes were of moderate severity, without symptoms, and no treatment was administrated. All side effects were transient and resolved spontaneously, with the exception of the case with subcutaneous induration, which persisted at the end of the study.

## Discussion

HIFU is an emerging noninvasive method of targeted thermal ablation. Hynynen et al. were the first to describe the results of magnetic resonance (MR)-guided HIFU treatment of breast FA, which was successful in 73% of the cases, with a significant decrease of the mean FA volume from 1.9 ml ± 1.5 (SD) to 1.3 ml ± 1.1 (*p* = 0.01) [17]. They concluded that the treatment was both feasible and safe without marked adverse events.

In this study, we have described the first follow-up of US-guided HIFU ablation of breast FA with significant volume reduction at 6 and 12 months after the treatment. Our results also demonstrated the advantages of US guidance of all treatment steps—visualization of the target, positioning, planning, and treatment control. The US imaging modality, using the same form of energy as for HIFU therapy, provided the benefit of a high-resolution and real-time image for planning and treatment control [16]. The visibility of HIFU-induced tissue changes as hyperechoic marks allowed monitoring of the treatment efficacy. MR has the advantage of temperature quantification and the ability to immediately evaluate the treatment [20]. However, MR guidance is expensive, labor intensive, and of lower spatial resolution in some cases [16].

Traditionally, symptomatic FA were treated by surgical excision [4]. However, there is increasing evidence that a conservative approach is safe and acceptable. A minority of FA will disappear without treatment, and the other lesions either increase in size or remain unchanged [6,21]. Clinically benign FA can be bothersome to some patients, causing physical deformity, discomfort, and/or emotional distress; most breast surgeons will comply with an informed patient’s preference for treatment, including traditional open surgical excision which is often performed for clinical symptoms or for the fear of malignancy [22].

Complications after surgery might be bleeding, wound infection, an unsatisfactory cosmetic result, and even recurrence. These side effects could compromise quality of life [3].

Regarding the benign nature of FA, an important treatment goal should be cosmesis [23]. The minimally invasive techniques as vacuum-assisted percutaneous excisional biopsy and percutaneous thermoablation with radiofrequency, or cryotherapy are promising for effective and safe treatment with acceptable cosmetic results [11,14,24]. However, Grady et al. reported up to 33% recurrence rate after vacuum-assisted excision of larger FA and longer follow-up [22], whereas US-guided HIFU is a completely noninvasive procedure, without skin incision and needle or probe insertion in the breast [17,19,25]. Scar formation and breast volume loss are avoided as side effects [23]. In 1-year follow-up, FA volume decreased or remained stable, without US data of regrowth. The rare cases of undiagnosed malignancy within a FA [26] should not be an argument against HIFU treatment, because the tumors are usually small, mainly in situ, and could be ablated together with the FA.

Another advantage of HIFU is the ability to reduce symptoms. Pain and discomfort related to FA at baseline were completely resolved at the time of the last assessment. Beyond the volume reduction, the most important aspect for the patient is the disappearance of FA perception, echoed by the signs and symptoms reduction. Patient satisfaction of the treatment was also demonstrated by the willingness of several patients to repeat the treatment for another FA in the same or in the contralateral breast.

Important factors in the global assessment of HIFU treatment are the hospital stay and the recovery duration. US-guided HIFU of breast FA is an outpatient procedure, performed under conscious sedation or perhaps with local anesthesia, and hence reduces costs incurred by surgical procedures with general anesthesia.

## Conclusions

We have shown the clinical outcome and safety of US-guided HIFU in patients with breast FA. Our results demonstrated that the method is effective in reducing the volume and clinical symptoms of FA without serious side effects. US-guided HIFU may become a noninvasive alternative to surgery. Further studies with longer follow-up are needed to establish the optimal treatment protocol and to assess the long-term efficacy.

## Abbreviations

FA: Fibroadenoma
CNB: Core needle biopsy
US: Ultrasound
HIFU: High-intensity focused ultrasound
VTU: Visualization treatment unit
VAS: Visual analog scale
MR: Magnetic resonance
